# Radiotherapy for metastatic spinal cord compression with increased radiation doses (RAMSES-01): a prospective multicenter study

**DOI:** 10.1186/s12885-019-6390-x

**Published:** 2019-11-29

**Authors:** Dirk Rades, Olfred Hansen, Lars Henrik Jensen, Liesa Dziggel, Christian Staackmann, Claudia Doemer, Jon Cacicedo, Antonio J. Conde-Moreno, Barbara Segedin, Raquel Ciervide-Jurio, Carmen Rubio-Rodriguez, Luis A. Perez-Romasanta, Ana Alvarez-Gracia, Kristopher Dennis, Carlos Ferrer-Albiach, Arturo Navarro-Martin, Fernando Lopez-Campos, Natalia Jankarashvili, Stefan Janssen, Denise Olbrich, Niels Henrik Holländer

**Affiliations:** 1 Department of Radiation Oncology, University of Lübeck, Ratzeburger Allee 160, 23562 Lübeck, Germany; 2 Department of Oncology, Odense University Hospital, Odense, Denmark; 3 Department of Oncology, University of Southern Denmark, Vejle, Denmark; 4 Department of Radiation Oncology, Cruces University Hospital, Barakaldo, Vizcaya Spain; 5 Department of Radiation Oncology, University and Polytechnic Hospital La Fe, Valencia, Spain; 6 Department of Radiotherapy, Institute of Oncology Ljubljana, Ljubljana, Slovenia; 7 Department of Radiation Oncology, University Hospital HM Sanchinarro, Madrid, Spain; 8 Department of Radiation Oncology, University Hospital Salamanca, Salamanca, Spain; 9 Department of Radiation Oncology, ICO - University Hospital Germans Trias i Pujol, Badalona, Spain; 10 Division of Radiation Oncology, The Ottawa Hospital & University of Ottawa, Ottawa, Canada; 11 Department of Radiation Oncology, Consorcio Hospital Provincial de Castellón, Castellón, Spain; 12 Department of Radiation Oncology, Catalan Institute of Oncology, L’Hospitalet de Llobregat, Barcelona, Spain; 13 Department of Radiation Oncology, University Hospital Ramón y Cajal, Madrid, Spain; 14 Department of Radiation Oncology, F. Todua Medical Center, Tbilisi, Georgia; 15 Medical Practice for Radiotherapy and Radiation Oncology, Hannover, Germany; 16 Centre for Clinical Trials Lübeck, Lübeck, Germany; 17 Department of Oncology and Palliative Units, Zealand University Hospital, Naestved, Denmark

**Keywords:** Metastatic spinal cord compression, Favorable survival prognosis, Precision radiotherapy, Increased radiation dose, Local progression-free survival

## Abstract

**Background:**

Patients with metastatic spinal cord compression (MSCC) and favorable survival prognoses can benefit from radiation doses greater than 30Gy in 10 fractions in terms of improved local progression-free survival (LPFS) and overall survival (OS).

**Methods/design:**

This prospective study mainly investigates LPFS after precision radiotherapy (volumetric modulated arc therapy or stereotactic body radiotherapy) with 18 × 2.33Gy in 3.5 weeks. LPFS is defined as freedom from progression of motor deficits during radiotherapy and an in-field recurrence of MSCC following radiotherapy. The maximum relative dose allowed to the spinal cord is 101.5% of the prescribed dose, resulting in an equivalent dose in 2Gy-fractions (EQD2) for radiation myelopathy is 45.5Gy, which is below the tolerance dose of 50Gy according to the Quantitative Analyses of Normal Tissue Effects in the Clinic (QUANTEC). The EQD2 of this regimen for tumor cell kill is 43.1Gy, which is 33% higher than for 30Gy in 10 fractions (EQD2 = 32.5Gy). Primary endpoint is LPFS at 12 months after radiotherapy. Secondary endpoints include the effect of 18 × 2.33Gy on motor function, ambulatory status, sensory function, sphincter dysfunction, LPFS at other follow-up times, overall survival, pain relief, relief of distress and toxicity. Follow-up visits for all endpoints will be performed directly and at 1, 3, 6, 9 and 12 months after radiotherapy. A total of 65 patients are required for the prospective part of the study. These patients will be compared to a historical control group of at least 235 patients receiving conventional radiotherapy with 10x3Gy in 2 weeks.

**Discussion:**

If precision radiotherapy with 18 × 2.33Gy results in significantly better LPFS than 10x3Gy of conventional radiotherapy, this regimen should be strongly considered for patients with MSCC and favorable survival prognoses.

**Trial registration:**

Clinicaltrials.gov NCT04043156. Registered 30-07-2019.

## Background

A considerable number of patients irradiated for metastatic spinal cord compression (MSCC) have a favorable survival prognosis with 6-month and 12-month survival rates of > 80 and > 70%, respectively [1, 2]. These patients are easily identified using validated prognostic tools [1, 2] and can live long enough to develop a recurrence of MSCC in the irradiated part of the spine. In case of such an in-field recurrence, many patients are not suitable for surgery [3, 4]. Moreover, safe administration of a second course of radiotherapy is often taking into account the risk of radiation myelopathy [5]. Longer-course radiotherapy programs (2–4 weeks) can result in better local control and local progression-free survival (LPFS) than short-course programs [6, 7]. In a retrospective matched-pair study, local control and LPFS were further improved with doses beyond the most commonly used longer-course regimen 30Gy in 10 fractions (10x3Gy) [8]. Increase of the dose for MSCC is limited by the radiation tolerance of the spinal cord [9, 10]. With precision radiotherapy techniques such as volumetric modulated arc therapy (VMAT) and stereotactic body radiotherapy (SBRT), radiation doses can be further increased than with conventional radiotherapy [11].

In the RAMSES-01 study, precision radiotherapy with 18 × 2.33Gy in 3.5 weeks is investigated. The equivalent dose in 2Gy-fractions (EQD2) of this regimen for tumor cell kill is 43.1Gy, which is 33% higher than for 30Gy in 10 fractions (32.5Gy) [12, 13]. The EQD2 of 18 × 2.33Gy for radiation myelopathy is 45.5Gy, which is below the tolerance dose of the spinal cord of 50Gy according to the Quantitative Analyses of Normal Tissue Effects in the Clinic (QUANTEC) [9]. The EQD2 of 18 × 2.33Gy for damage to the vertebral bone is 45.1Gy, which is below the tolerance dose of bone of 52Gy [9, 10]. Thus, precision radiotherapy with 18 × 2.33Gy can be considered safe.

This study includes two parts, a single-arm trial of patients receiving 18 × 2.33Gy and a comparison of this cohort to a historical control group treated with 10x3Gy. It aims to show that 18 × 2.33Gy of precision radiotherapy results in significantly better LPFS than 10x3Gy of conventional RT. If such superiority is shown, 18 × 2.33Gy could be recommended for patients with favorable survival prognoses.

## Methods/design

### Endpoints of the study

The primary endpoint is the 12-month LPFS following 18 × 2.33Gy of VMAT (preferred) or SBRT (possible for a single vertebra) in patients with favorable survival prognoses according to a validated score [1, 2]. This survival score is used by many physicians worldwide when aiming to assign the appropriate radiation regimen to a patient with MSCC.

### Study design

The first part of this study represents a single-arm trial and evaluates the effect of precision radiotherapy with 18 × 2.33Gy given over 3.5 weeks on LPFS. Sixty-five patients (62 patients + 5% for drop-outs) are supposed to be recruited within 21 months. The characteristics to be recorded to allow a comparison with the historical control group include age, gender, primary tumor type, interval between tumor diagnosis and MSCC, number of vertebrae affected by MSCC, additional bone or visceral metastases, time developing motor deficits, pre-radiotherapy ambulatory status, and performance status according to the Eastern Cooperative Oncology Group (ECOG) [7]. Propensity score techniques will be applied to reduce confounding due to differences between the historical control group and prospective trial data [14]. The inclusion and exclusion criteria are almost identical to those of a previous trial investigating 5x5Gy of precision radiotherapy of MSCC [11]. Only the inclusion criteria are supplemented by favorable survival prognosis (defined as 36–45 points on a survival score) [1, 2].

### Treatment

Radiotherapy is administered with VMAT (or SBRT) with 2.33Gy per fraction up to 42.0Gy in 3.5 weeks. This regimen represents an EQD2 of 43.1Gy for tumor cell kill, which means an increase of the radiation dose by 33% compared to 10x3Gy in 2 weeks (EQD2 = 32.5Gy). The EQD2 for radiation myelopathy is 45.5Gy for 100% of the prescribed dose [12, 13]. An EQD2 of <50Gy is considered safe and estimated to be associated with a risk of radiation-related myelopathy of < 0.2% [9]. Treatment should be started as soon as possible, i.e. within 48 h after first presentation to a radiation oncologist.

The planning target volume (PTV) should include the involved vertebrae plus 1 cm above and below. The PTV should be covered by the 95%-isodose. The spinal cord should not receive more than 101.5% of the prescribed dose (EQD2 = 46.6Gy for radiation myelopathy, α/β = 2Gy). This maximum dose is estimated to be associated with a risk of radiation-related myelopathy of < 0.2% [9]. Both the EQD2 of the prescribed dose (45.1Gy) and the EQD2 of the maximum dose (46.1Gy, α/β = 2.5Gy) are below the tolerance dose of bone of 52Gy [9, 11]. The mean doses (EQD2) for esophagus, heart and lung must be <34Gy, <26Gy and ≤ 7Gy [9]. The patients should receive concomitant corticosteroids during radiotherapy [15, 16].

### Assessments

The following endpoints will be prospectively assessed by the participating physicians directly and at 1, 3, 6, 9 and 12 months after radiotherapy and recorded in a case report form (CRF): Motor function, ability to walk, sensory function, sphincter dysfunction, LPFS, overall survival (OS), pain relief, relief of distress, and toxicity. If a recurrence of MSCC is clinically suspected (deterioration of motor function following improvement or no change of motor function during radiotherapy), MRI will be performed. For MRI, rates of sensitivity, specificity and diagnostic accuracy regarding the detection of MSCC of 93, 97, and 95%, respectively, were reported [16, 17]. In case of an out-field recurrence of MSCC, the patient will be censored for LPFS. Assessment directly after radiotherapy will result in a difference of one and a half week between the prospective cohort and the historical control group. However, this way of assessment was selected, since the primary endpoint LPFS included no progression of motor deficits during radiotherapy (=immediate response), which would ideally be assessed directly after the end of radiotherapy. Motor function will be evaluated with a 5-point scale [11, 18]. Sensory function will be assessed as absent, impaired or normal, sphincter dysfunction as yes or no [19]. For assessment of pain, a numeric self-assessment scale will be used (0–10 points) [20]. Distress will be evaluated with the distress thermometer (0–10 points [21, 22]. For assessment of toxicity, the Common Terminology Criteria for Adverse Events version 4.03 will be used [23].

### Comparisons with the historical control group

The patients receiving 18 × 2.33Gy will be compared to historical control group of patients with a favorable survival prognosis treated with 10x3Gy of conventional radiotherapy from an anonymized database. Patients of the control group must fulfil the same inclusion and exclusion criteria as the patients of the prospective part of the study. It is estimated that 235 patients will qualify for the control group. To reduce the risk of hidden selection biases, a propensity score approach including 10 potential prognostic factors will be used for comparisons between the prospective cohort and the historical control group [7, 11].

### Sample size calculation

The primary aim is to evaluate the LPFS at 12 months after 18 × 2.33Gy using VMAT or SBRT and to show superiority to 10x3Gy of conventional radiotherapy.

With respect to tumor cell kill, the EQD2 of 18 × 2.33Gy is considerably higher (+ 33%) than the EQD2 of 10x3Gy (43.1Gy vs. 32.5Gy). In a previous study, the 12-month LPFS rate was 84% with 10x3Gy in 2 weeks [8]. An increase by 12.5 percentage points is considered clinically important. Sixty-two eligible patients are required for estimation of the 12-month LPFS with appropriate precision. The statistical power should be at least 80%. Assuming that 5% of the patients will not be eligible for the efficacy analysis, a total of 65 patients should be recruited for the prospective trial.

For the comparison of the prospective trial and the historical cohort group, propensity score methods will be used to reduce confounding due to differences between the two data sets. Assuming that this comparison is performed with a simple Pearson-Chi-Square test (two-sided significance level = 5%), the power will be 77.9%, if the data of 62 prospectively treated patients and the data of 235 patients serving as historical control group can be used. Since the historical control database is constantly growing, the power will likely be 80% or higher at the time of the final analyses.

### Data management

All data relating to patients will be recorded in a pseudonymous way. Each patient will be identifiable only by the unique patient number, date of birth and gender. A patient identification list will only be kept in the relevant study centers and will not be forwarded to the sponsor. Data collection will be done using the paper-based case report forms. These forms should be filled in as soon as possible and be submitted to the checker for review, signed, dated and forwarded to the study management via fax or secure email.

The originals of all key study documents, including the documentation sheets, will be kept at the study headquarters for a minimum of 10 years after the final report. The principal investigator/head of the study center will keep all administrative documents (written correspondence with the ethics committee, regulatory authorities, study management, study headquarters), the patient identification list, the signed informed consent forms, copies of the documentation sheets and the general study documentation (protocol, amendments) for the above mentioned period. Original patient data (patient files) must also be kept for the length of time stipulated for the study centres, but not for less than 10 years. The site principle investigators are responsible for the day-to-day organization and the data management at their sites.

## Discussion

Despite an increasing use of upfront decompressive surgery in addition to radiotherapy, the majority of patients with MSCC still receive radiotherapy alone [3, 4, 15, 16]. Short-course radiotherapy programs such as 5x4Gy within 1 week are not inferior to longer-course programs such as 10x3Gy with respect to the effect on motor function and ambulatory status [24, 25]. However, longer-course programs result in better local control of MSCC and LPFS, particularly in patients with favorable survival prognoses [6, 7, 24]. In a prospective non-randomized trial of patients with MSCC and poor to favorable survival prognoses, the 1-year local control rates were 81% after longer-course and 61% after short-course radiotherapy (*p* = 0.005) [6]. Patients with favorable prognoses are at a higher risk to experience an in-field recurrence of MSCC, since the risk of such a recurrence increases with survival time. Moreover, a retrospective study of patients with favorable survival prognoses (according to a survival score that has been validated in a prospective cohort of patients) suggested that these patients can benefit from radiation doses beyond 30Gy in 10 fractions [1, 2, 8]. In that study, 191 patients receiving 30Gy in 10 fractions were matched to 191 patients treated with 37.5Gy in 15 fractions or 40Gy in 20 fractions [8]. In order to reduce the risk of a hidden selection bias, the patients were matched 1:1 for 10 characteristics including age, gender, tumor type, performance status, number of involved vertebrae, visceral metastases, other bone metastases, interval from tumor diagnosis to radiotherapy, ambulatory status, and time developing motor deficits. Patients receiving 37.5Gy or 40Gy did achieve better outcomes in terms of local control of MSCC (92% vs. 71% at 2 years, *p* = 0.012), LPFS (90% vs. 68%, *p* = 0.013) and OS (68% vs. 53%, *p* = 0.032).

One important question is whether outcomes of radiotherapy for MSCC in patients with favorable survival prognoses can be further increased with radiation doses beyond 40Gy. LPFS is an important endpoint, since a lack of response to radiotherapy and an in-field recurrence of MSCC associated with neurologic deficits must be considered serious for the patients. For many of these patients, decompressive surgery is not possible. Moreover, in case of an in-field recurrence, a second radiation course may lead to exceedence of the tolerance dose of the spinal cord resulting in radiation myelopathy with severe neurologic deficits [9, 10]. The effect of radiotherapy on motor function was not selected as primary endpoint, since a previous retrospective study in patients with MSCC and favorable survival prognoses suggested a benefit of higher radiation doses regarding LPFS but not regarding post-treatment motor function [8]. Moreover, the previous randomized trials of radiotherapy for MSCC that were not limited to patients with favorable prognoses did not show a benefit for higher doses with respect to improvement of motor function, which is particularly important for patients with poor or intermediate survival prognoses who likely will not live long enough to experience a local recurrence of MSCC [25–29].

Increasing the radiation dose in order to improve LPFS is also limited due to the tolerance dose of the spinal cord [9, 10]. With conventional radiotherapy, the maximum dose to the spinal cord is always higher than 100% (frequently about 105%) of the prescribed dose, which accounts for both total dose and dose per fraction, resulting in a significantly higher EQD2 for myelopathy. In a previous trial of precision radiotherapy for MSCC, the maximum dose to the spinal cord could be reduced to 101.5% [11]. The same constraint is used for the present RAMSES-01 trial. This allows safe administration of a radiation dose higher than 40Gy (EQD2 = 43.1Gy). The dose administered in the RAMSES-01 trial represents an increase of the EQD2 for tumor cell kill by 33% when compared to 10x3Gy, the most commonly used longer-course program for MSCC worldwide. In a previous prospective study of precision radiotherapy for MSCC, radiation treatment could be delivered within 24 h [11]. Thus, the use of precision radiotherapy did not delay treatment.

A higher EQD2 can also be administered with single-fraction SBRT. However, the tolerance doses of spinal cord and vertebral bone must be taken into account to avoid neurologic deficits and vertebral fractures [30, 31]. The updated ASTRO evidence-based guideline recommends that SBRT for MSCC should be limited to clinical trials [32]. Since single-fraction SBRT with ≥20Gy has been identified as a significant risk factor for vertebral fractures, fractionated precision radiotherapy (SBRT or VMAT) is considered a preferable option [33, 34].

If this new approach of precision radiotherapy with 18 × 2.33Gy proves to be superior to 10x3Gy of conventional radiotherapy for LPFS, this regimen should be strongly considered for patients with MSCC and favorable survival prognoses.

## Data Availability

The study has been registered at clinicaltrials.gov (identifier: NCT04043156), where data regarding the study are available as well.

## Abbreviations

CT: Computed tomography
CTCAE: Common Terminology Criteria for Adverse Events
ECOG: Eastern Cooperative Oncology Group
EQD2: Equivalent dose in 2Gy-fractions
LPFS: Local progression-free survival
MRI: Magnetic resonance imaging
MSCC: Metastatic spinal cord compression
OS: Overall survival
PTV: Planning target volume
QUANTEC: Quantitative Analyses of Normal Tissue Effects in the Clinic
SBRT: Stereotactic body radiotherapy
VMAT: Volumetric modulated arc therapy

## References

[1] Rades D, Dunst J, Schild SE (2008). The first score predicting overall survival in patients with metastatic spinal cord compression. Cancer.

[2] Rades D, Douglas S, Veninga T, Stalpers LJ, Hoskin PJ, Bajrovic A, Adamietz IA, Basic H, Dunst J, Schild SE (2010). Validation and simplification of a score predicting survival in patients irradiated for metastatic spinal cord compression. Cancer.

[3] Patchell R, Tibbs PA, Regine WF, Payne R, Saris S, Kryscio RJ, Mohiuddin M, Young B (2005). Direct decompressive surgical resection in the treatment of spinal cord compression caused by metastatic cancer: a randomised trial. Lancet.

[4] Rades D, Huttenlocher S, Dunst J, Bajrovic A, Karstens JH, Rudat V, Schild SE (2010). Matched pair analysis comparing surgery followed by radiotherapy and radiotherapy alone for metastatic spinal cord compression. J Clin Oncol.

[5] Nieder C, Grosu AL, Andratschke NH, Molls M (2006). Update of human spinal cord reirradiation tolerance based on additional data from 38 patients. Int J Radiat Oncol Biol Phys.

[6] Rades D, Lange M, Veninga T, Stalpers LJ, Bajrovic A, Adamietz IA, Rudat V, Schild SE (2011). Final results of a prospective study comparing the local control of short-course and long-course radiotherapy for metastatic spinal cord compression. Int J Radiat Oncol Biol Phys.

[7] Rades D, Fehlauer F, Schulte R, Veninga T, Stalpers LJ, Basic H, Bajrovic A, Hoskin PJ, Tribius S, Wildfang I, Rudat V, Engenhart-Cabilic R, Karstens JH, Alberti W, Dunst J, Schild SE (2006). Prognostic factors for local control and survival after radiotherapy of metastatic spinal cord compression. J Clin Oncol.

[8] Rades D, Panzner A, Rudat V, Karstens JH, Schild SE (2011). Dose escalation of radiotherapy for metastatic spinal cord compression (MSCC) in patients with relatively favorable survival prognosis. Strahlenther Onkol.

[9] Marks LB, Yorke ED, Jackson A, Ten Haken RK, Constine LS, Eisbruch A, Bentzen SM, Nam J, Deasy JO (2010). Use of normal tissue complication probability models in the clinic. Int J Radiat Oncol Biol Phys.

[10] Emami B (2013). Tolerance of the normal tissue to therapeutic irradiation. Rep Radiother Oncol.

[11] Rades D, Cacicedo J, Conde-Moreno AJ, Doemer C, Dunst J, Lomidze D, Segedin B, Olbrich D, Holländer NH (2017). High-precision radiotherapy of motor deficits due to metastatic spinal cord compression (PRE-MODE): a multicenter phase 2 study. BMC Cancer.

[12] Barendsen GW (1982). Dose fractionation, dose rate and iso-effect relationships for normal tissue responses. Int J Radiat Oncol Biol Phys.

[13] Joiner MC, Van der Kogel AJ, Steel GG (1997). The linear-quadratic approach to fractionation and calculation of isoeffect relationships. Basic clinical radiobiology.

[14] Rosenbaum PR, Rubin DB (1983). The central role of the propensity score in observational studies for causal effects. Biometrika.

[15] Prasad D, Schiff D (2005). Malignant spinal cord compression. Lancet Oncol.

[16] Rades D, Abrahm JL (2010). The role of radiotherapy for metastatic epidural spinal cord compression. Nat Rev Clin Oncol.

[17] Li KC, Poon PY (1988). Sensitivity and specificity of MRI in detecting malignant spinal cord compression and in distinguishing malignant from benign compression fractures of vertebrae. Magn Reson Imaging.

[18] Tomita T, Galicich JH, Sundaresan N (1983). Radiation therapy for spinal epidural metastases with complete block. Acta Radiol Oncol.

[19] Curt A, Dietz V (1997). Zur Prognose traumatischer Rückenmarkläsionen. Nervenarzt.

[20] Chow E, Hoskin P, Mitera G, Zeng L, Lutz S, Roos D, Hahn C, van der Linden Y, Hartsell W, Kumar E (2012). International bone metastases consensus working party: update of the international consenus on palliative radiotherapy endpoints for future clinical trials in bone metastases. Int J Radiat Oncol Biol Phys.

[21] Holland JC (1999). Update: NCCN practice guidelines for the management of psychosocial distress. Oncology.

[22] Holland JC, Andersen B, Breitbart WS, Buchmann LO, Compas B, Deshields TL, Dudley MM, Fleishman S, Fulcher CD, Greenberg DB, Greiner CB, Handzo GF, Hoofring L, Hoover C, Jacobsen PB, Kvale E, Levy MH, Loscalzo MJ, McAllister-Black R, Mechanic KY, Palesh O, Pazar JP, Riba MB, Roper K, Valentine AD, Wagner LI, Zevon MA, McMillian NR, Freedman-Cass DA (2013). Distress management. J Natl Comp Cancer Netw.

[23] National Institutes of Health/National Cancer Institute (2010). Common Terminology Criteria for Adverse Events (CTCAE) version 4.03.

[24] Rades D, Stalpers LJ, Veninga T, Schulte R, Hoskin PJ, Obralic N, Bajrovic A, Rudat V, Schwarz R, Hulshof MC, Poortmans P, Schild SE (2005). Evaluation of five radiation schedules and prognostic factors for metastatic spinal cord compression. J Clin Oncol.

[25] Rades D, Šegedin B, Conde-Moreno AJ, Garcia R, Perpar A, Metz M, Badakhshi H, Schreiber A, Nitsche M, Hipp P, Schulze W, Adamietz IA, Norkus D, Rudat V, Cacicedo J, Schild SE (2016). Radiotherapy with 4 Gy × 5 versus 3 Gy × 10 for metastatic epidural spinal cord compression: final results of the SCORE-2 trial (ARO 2009/01). J Clin Oncol.

[26] Maranzano E, Bellavita R, Rossi R, De Angelis V, Frattegiani A, Bagnoli R, Mignogna M, Beneventi S, Lupattelli M, Ponticelli P, Biti GP, Latini P (2005). Short-course versus split-course radiotherapy in metastatic spinal cord compression: results of a phase III, randomized, multicenter trial. J Clin Oncol.

[27] Maranzano E, Trippa F, Casale M, Costantini S, Lupattelli M, Bellavita R, Marafioti L, Pergolizzi S, Santacaterina A, Mignogna M, Silvano G, Fusco V (2009). 8Gy single-dose radiotherapy is effective in metastatic spinal cord compression: results of a phase III randomized multicentre Italian trial. Radiother Oncol.

[28] Thirion P, O’Sullivan L, Clayton-Lea A, Small C, McArdle O, Kelly P, Parker I, O'Sullivan J, Hacking D, Collins C, Pomeroy M, Moriarty M (2014). ICORG 05–03: Prospective randomized non-inferiority phase 3 trial comparing two radiation schedules in malignant spinal cord compression not proceeding with surgical decompression. Int J Radiat Oncol Biol Phys.

[29] Hoskin P, Misra V, Hopkins K, Holt T, Brown G, Arnott S, Thomas SS, Reczko K, Beare S, Lopes A, Forsyth S (2017). SCORAD III: Randomized noninferiority phase III trial of single-dose radiotherapy (RT) compared to multifraction RT in patients (pts) with metastatic spinal canal compression (SCC). J Clin Oncol.

[30] Garg AK, Shiu AS, Yang J, Wang XS, Allen P, Brown BW, Grossman P, Frija EK, McAleer MF, Azeem S, Brown PD, Rhines LD, Chang EL (2012). Phase 1/2 trial of single-session stereotactic body radiotherapy for previously unirradiated spinal metastases. Cancer.

[31] Chang JH, Shin JH, Yamada YJ, Mesfin A, Fehlings MG, Rhines LD, Sahgal A (2016). Stereotactic body radiotherapy for spinal metastases: What are the risks and how do we minimize them?. Spine.

[32] Lutz S, Balboni T, Jones J, Lo S, Petit J, Rich SE, Wong R, Hahn C (2017). Palliative radiation therapy for bone metastases: update of an ASTRO evidence-based guideline. Pract Radiat Oncol.

[33] Faruqi S, Tseng CL, Whyne C, Alghamdi M, Wilson J, Myrehaug S, Soliman H, Lee Y, Maralani P, Yang V, Fisher C, Sahgal A (2018). Vertebral compression fracture after spine stereotactic body radiation therapy: a review of the pathophysiology and risk factors. Neurosurgery.

[34] Guckenberger M, Mantel F, Gerszten PC, Flickinger JC, Sahgal A, Létourneau D, Grills IS, Jawad M, Fahim DK, Shin JH, Winey B, Sheehan J, Kersh R (2014). Safety and efficacy of stereotactic body radiotherapy as primary treatment for vertebral metastases: a multi-institutional analysis. Radiat Oncol.

